# Metabolomics Data Normalization with EigenMS

**DOI:** 10.1371/journal.pone.0116221

**Published:** 2014-12-30

**Authors:** Yuliya V. Karpievitch, Sonja B. Nikolic, Richard Wilson, James E. Sharman, Lindsay M. Edwards

**Affiliations:** 1 School of Mathematics and Physics, University of Tasmania, Hobart, TAS, Australia; 2 Menzies Research Institute Tasmania, University of Tasmania, Hobart, TAS, Australia; 3 Central Science Laboratory, University of Tasmania, Hobart, TAS, Australia; 4 Centre of Human & Aerospace Physiological Sciences, King’s College London, London, United Kingdom; 5 Fibrosis Discovery Performance Unit, GlaxoSmithKline R&D, Stevenage, United Kingdom; University of Westminster, United Kingdom

## Abstract

Liquid chromatography mass spectrometry has become one of the analytical platforms of choice for metabolomics studies. However, LC-MS metabolomics data can suffer from the effects of various systematic biases. These include batch effects, day-to-day variations in instrument performance, signal intensity loss due to time-dependent effects of the LC column performance, accumulation of contaminants in the MS ion source and MS sensitivity among others. In this study we aimed to test a singular value decomposition-based method, called EigenMS, for normalization of metabolomics data. We analyzed a clinical human dataset where LC-MS serum metabolomics data and physiological measurements were collected from thirty nine healthy subjects and forty with type 2 diabetes and applied EigenMS to detect and correct for any systematic bias. EigenMS works in several stages. First, EigenMS preserves the treatment group differences in the metabolomics data by estimating treatment effects with an ANOVA model (multiple fixed effects can be estimated). Singular value decomposition of the residuals matrix is then used to determine bias trends in the data. The number of bias trends is then estimated via a permutation test and the effects of the bias trends are eliminated. EigenMS removed bias of unknown complexity from the LC-MS metabolomics data, allowing for increased sensitivity in differential analysis. Moreover, normalized samples better correlated with both other normalized samples and corresponding physiological data, such as blood glucose level, glycated haemoglobin, exercise central augmentation pressure normalized to heart rate of 75, and total cholesterol. We were able to report 2578 discriminatory metabolite peaks in the normalized data (p<0.05) as compared to only 1840 metabolite signals in the raw data. Our results support the use of singular value decomposition-based normalization for metabolomics data.

## Introduction

Along with nuclear magnetic resonance, liquid chromatography coupled to mass spectrometry (LC-MS) has become one of the most common analytical platforms for studying cell, tissue or body fluid metabolomes [1]–[7]. Advantages of the method include high sensitivity and the ability to discriminate thousands of features in a single experiment. Yet, as with any high-throughput technology, systematic biases are often observed in LC-MS metabolomics data [8]. As the number of samples in the dataset increases so does the possibility of a time-dependent variation in the resulting metabolite data. Time-dependent trends in LC-MS metabolomics datasets typically result from analyte retention time drift due to changes in LC column performance or variations in signal intensity caused by fluctuations in MS sensitivity. While these issues can be addressed in part by careful experimental design and the use of quality control samples, there remains a need for robust post-acquisition data normalization. Normalization methods need to be flexible enough to capture biases of arbitrary complexity, while avoiding overfitting that would invalidate downstream statistical inference. Careful normalization of metabolite peak intensities enables greater accuracy in quantitative comparisons between disease groups as well as better correlation of metabolite signals to physiological or phenotypic data collected in tandem. We report here the application of a singular value decomposition-based method, called EigenMS, to remove systematic biases from metabolomics data in the presence of missing observations [9]. This normalization method, previously shown to be effective in normalizing LC-MS proteomics data [9], improved downstream differential analysis and increased correlation of the metabolite peak intensities with corresponding physiological measurements of what we call clinical biochemistry.

## Materials and Methods

To demonstrate the utility of our approach, we used a recently-acquired metabolomic dataset examining the serum of subjects with type 2 diabetes (n = 40) and control subjects without diabetes (n = 39). The age range of the human subjects was 57±11 years with standard error of the mean of 1.2. Biological sample preparation and data acquisition followed the same protocol as reported in Nikolic et al. [10]. LC-MS data were acquired in positive ion mode using an Orbitrap XL mass spectrometer (Thermo Scientific) controlled by XCalibur v2 software. Chromatographic separation was carried out using C18 reverse-phase HPLC as described in [8].

The study was approved by the Human Research Ethics Committee of the University of Tasmania and all procedures conformed to the Declaration of Helsinki. Study participants gave written informed consent prior to participation.

We generated a pooled quality control (QC) sample in order to monitor LC and MS performance across sample runs by combining small aliquots (10 uL) of every sample in the study, as recommended by Sangster et al. [11]. This pooled QC sample was then used throughout the experiment as a process control. Because all QC injections originated from the same mixture and thus should be chemically identical, QC samples allow detection of variations in the observed intensities that may affect downstream statistical analyses.

We monitored system performance using blocks of four experimental samples flanked by the QC samples between MS source and inlet cleaning. Thus our basic experimental running order included cleaning of the ion spray cone and exterior surface of the ion transfer capillary with 50∶50 methanol/water between each block. At the end of each day of operation we used 50∶50 methanol/water to flush the sample transfer tube and atmospheric pressure ionization probe, according to the guidelines for daily operation of LTQ XL specified by the manufacturer.

Mass spectral peak deconvolution and retention time correction were carried out in R using XCMS. The parameters for deconvolution were: method = ‘centWave’, ppm = 3, peakwidth = 5–30, snthresh = 6, mzdiff = 0.01; and for RT correction: method = “obiwarp” and profStep = 0.1, yielding ∼7000 peaks.

EigenMS uses a combination of ANOVA and singular value decomposition to capture and remove biases from LC-MS metabolomic peak intensity measurements while preserving the variation of interest. ANOVA is used first to capture and preserve the variation attributable to the treatment effect(s) under study. EigenMS utilizes built-in R function lm() to estimate treatment effects and produce the matrix of residuals which accessed via the residuals() function. Singular value decomposition (SVD) is then applied to a matrix of residuals to find any systematic trends attributable to bias. The number of bias trends is determined by a permutation test and the effects of the bias trends are then removed from the data. We found that in metabolomic studies the number of bias trends to be eliminated should be set to about 20% of the number of samples. This is a heuristic approach that produces better normalization than the automatically determined number, which works well for proteomic studies. EigenMS is based on the surrogate variable analysis of Leek and Storey [12], with modifications including analysis of data with missing values that are typical in LC-MS experiments [9]. EigenMS is available as a stand-alone set of two functions implemented in R from SourceForge: http://sourceforge.net/projects/eigenms/. Current version of software was implemented in RStudio version 0.98.953 and R version 3.0.1.

## Results and Discussion

A characteristic of biofluid metabolite analysis via LC-MS is progressive signal intensity loss due to the accumulation of contaminants within the MS ion source, sample transfer lines and the heated ion transfer capillary [13]. We designed our experiment based on the guidelines outlined in Lai et al. 2009 [13] as well as based on in-house experimentation to establish the number of samples that could be run without large signal intensity loss on our instrumentation. We conditioned the LC column using QC samples as suggested by Want *et al.* and others prior to running any experimental samples to avoid high signal variation during the first few runs [8], [11].

**Figure 1:**
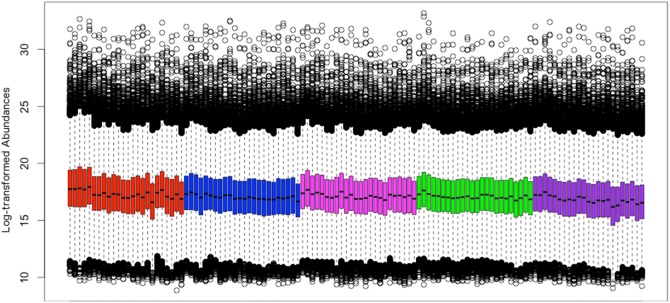
Boxplots of sample intensities. Boxplots of sample intensities in run order on the instrument for positive ion mode. Each box represents a sample, each of five days is presented in different color. Decline in intensity indicates signal intensity loss within each day.

Within each block, different treatment groups were matched and run order randomized. However, even using frequent cleaning, some signal loss was observed as is evident from the declining abundance profile within each day (Fig. 1). These data were acquired on the instrument operating in positive ion mode. Intensity loss was not as obvious when the instrument was operating in negative ion mode, however, we still observed variation that could not be easily explained. Careful experimental design, such as within-block treatment group matching, alleviated the influence of some of the biases and signal intensity loss that we and others have observed [13]. We still suggest that normalization be used to correct for any remaining intensity loss as well as any other known and unknown systematic biases. Further, if one wishes to identify relationships between LC-MS metabolomics data and other variables, normalization becomes essential.

Sample intensity loss makes comparisons between experimental groups more difficult, it also leads to an increased number of missing values in subsequent samples. Thus careful experimental design and sample run order randomization are required to minimize the introduction of systematic biases and any possible confounding of the results. Our experience leads us to recommend the use of QC samples to monitor performance of the LC-MS instrument. For example, we embedded QC samples throughout the experimental runs to help us monitor instrument performance. We utilized an incomplete block design where each block consisted of four samples, two diabetes and two control samples in randomized order. Each block was bracketed by QC samples to allow the operator to monitor the experiment and perform system diagnostics if variation in the signal intensity of the QC runs was observed. In this study QC samples were not used for data processing or normalization.

**Figure 2:**
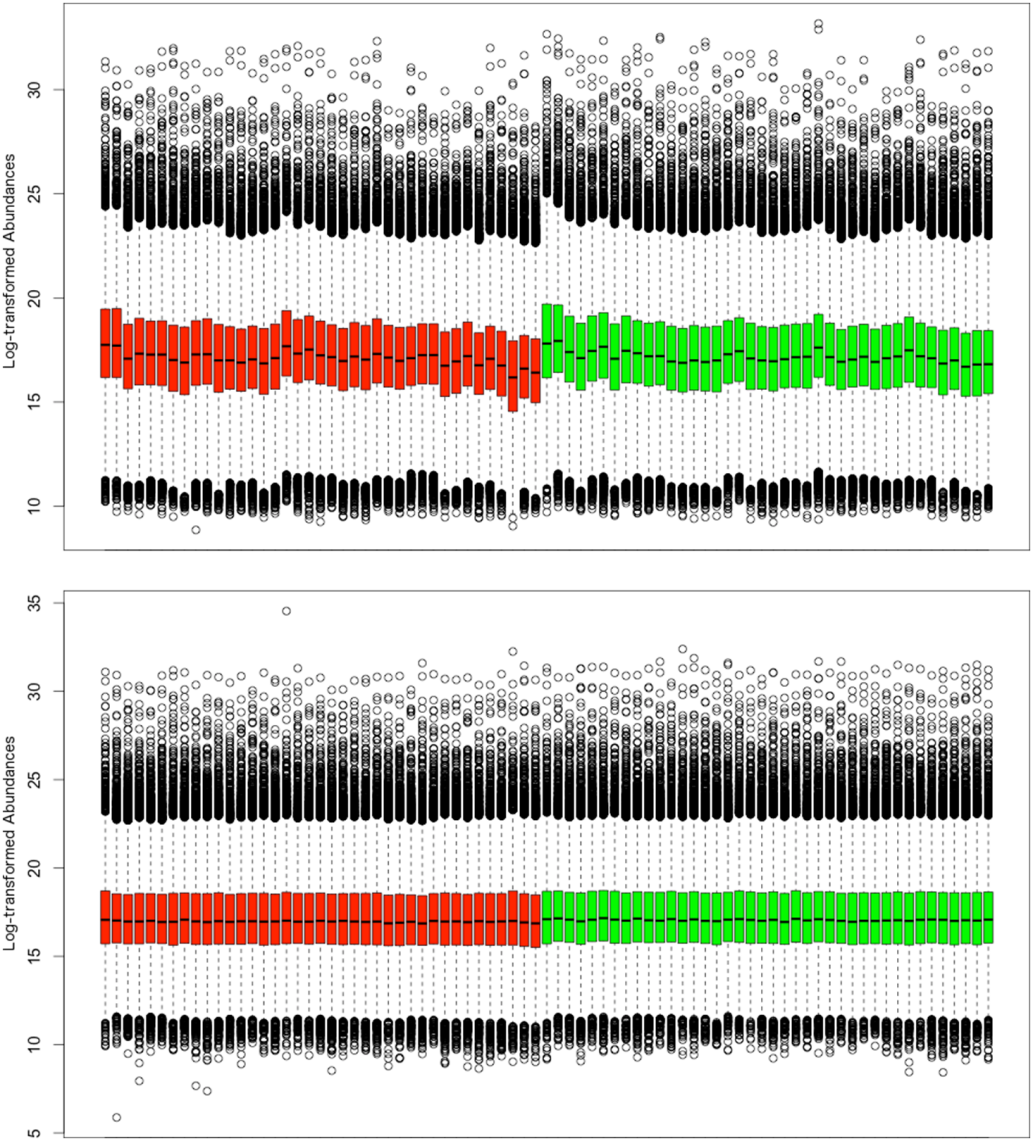
Boxplots of the raw and normalized intensities. Boxplots of the intensities before (top panel) and after (bottom panel) normalization. Each box represents a sample. Samples are grouped by disease group (red vs. green, QC samples are omitted) and are in chronological run order on the instrument within each group.

We normalized the data with EigenMS. EigenMS identified 12 systematic bias trends and eliminated their effects from the data. Fig. 2 shows boxplots of the intensities for the disease and control groups before (top panel) and after (bottom panel) normalization for the same data as in Fig. 1. Samples in Fig. 2 are grouped by disease group (red vs. green with QC samples not shown) and within each group they appear in the run order on the instrument, such that the first sample in red was run right next to the first sample in green and so on. Even with regular cleaning of the inlet we encountered some signal loss as is evident from the downward trend in the means (middle bars) of the boxplots. Fig. 2 bottom panel shows that normalization successfully adjusted for the signal intensity loss and any other systematic biases and placed the means of each sample almost on a straight line.

**Figure 3:**
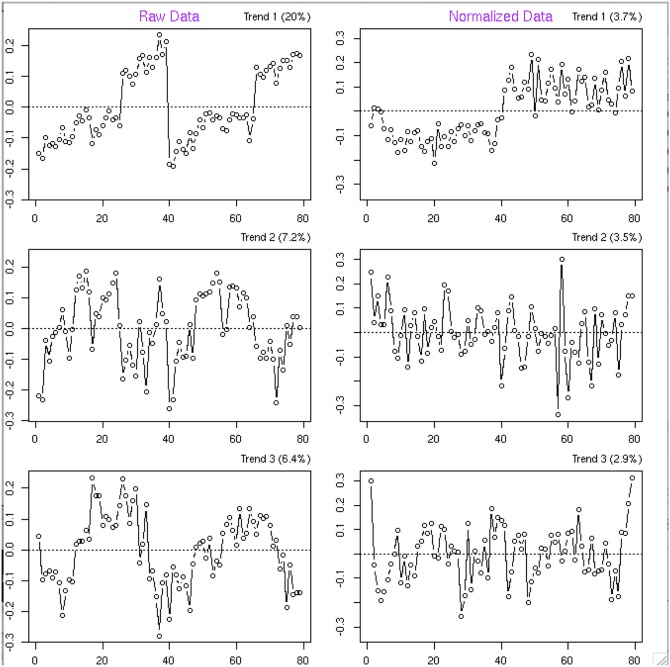
SVD trends in raw and normalized data. SVD trends in raw (left panel) and normalized (right panel) clinical study data. Percentage at the top of each subplot shows the percent of the variation in the data explained by each trend. On the *x*-axis is sample index from 1 to 79, where each circle represents a sample. Samples are grouped by disease group with 39 control samples numbered 1**–**39 followed by 40 diabetes samples numbered 40**–**79. Samples are in chronological run order on the instrument within each group. Values on the y-axis satisfy equation: *R* = *UDV*’ where *R* is a matrix of residuals (left panel) and matrix of normalized intensities (right panel); columns of *V* represent trends observed in the data. Three trends that explain the most amount of variation are plotted for raw and normalized data.

Fig. 3 shows SVD trends in raw (left 3 panels) and normalized (right 3 panels) diabetes data. Samples are ordered by group and appear in chronological run order within each group in the same way as Fig. 2. Trends are ordered from top to bottom by the decreasing amount of variation explained by each trend. Due to the nature of SVD, each trend is orthogonal to every other. Notably, 20 percent of the variation in the raw data is attributable to the signal loss that appears as the top trend. Note that SVD trends can be rotated around the x-axis, thus the top trend in the raw data represents signal intensity loss. The top trend also shows a jump in each group which occurred between days 3 and 4 of the experiment. All the processing was done following the same protocol, but we still observed a variation due to the day effect where samples run on days 4 and 5 were affected differently than samples run on days 1–3. The rest of the trends in the raw data are not easily interpretable and are attributed to unknown systematic biases. Fig. 3 (right panel) shows the normalized data. The top trend is representative of the differences between the disease and control groups. Only 3.7% of variation is attributable to the differences between the disease groups, but nonetheless this is a major trend in the normalized data as compared to the signal intensity loss trend in the raw data. Most importantly we were able to report 2578 discriminatory metabolite peaks in the normalized data as compared to only 1840 metabolite signals in the raw data (compared using an unpaired t-test; with a significance cutoff α = 0.05 after Benjamini-Hochberg multiple testing adjustment) [14].

We saw an improvement in correlations of normalized metabolite intensities to the physiology variables we measured for each subject. We selected 1100 peaks that were found to be significantly different between two groups with Benjamini-Hochberg adjusted p-value<0.001 in the normalized data. We correlated these 1100 MS peaks using Spearman correlation in ‘Hmisc’ package to the following clinical measurements we obtained for each subject: blood glucose level, glycated haemoglobin, exercise central augmentation pressure normalized to heart rate of 75, and total cholesterol [15]. The first three of these are expected to be elevated in diabetics while cholesterol is usually lower than controls due to statin therapy. If EigenMS works well we expect to see increased correlation between the changes observed in normalized metabolites and physiological variables.

**Figure 4:**
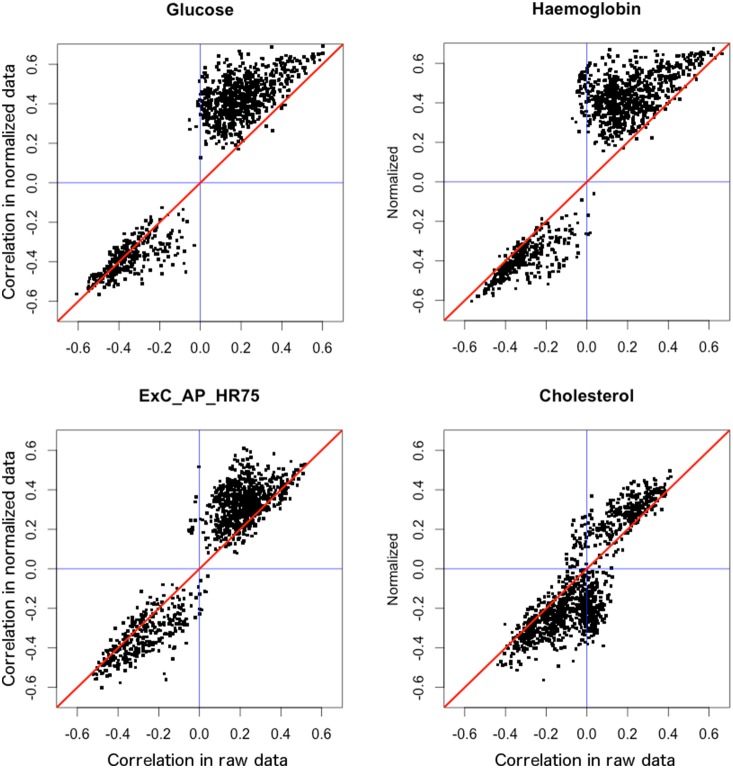
Correlations to physiology data. Correlations of raw (x-axis) and normalized (y-axis) metabolite intensities to physiology data. Metabolites with p-value<0.001 in the normalized data (1100 metabolites) were selected to be plotted. The same metabolites are plotted for raw data. Blue lines represent correlation of zero. Red diagonal line shows the line on which all dots would fall if there was no difference between correlations of raw and normalized data. Observed counter-clockwise shift of points indicates better correlation with physiological data.

Fig. 4 shows correlations of raw (x-axis) and normalized (y-axis) metabolite intensities to glucose (top left), glycated haemoglobin (top right), exercise central augmentation pressure (bottom left) and cholesterol (bottom right). The blue lines show correlations of zero. The red lines indicate no difference between correlations obtained from normalized and raw data. The scatter plots show that we obtained higher correlations of normalized metabolite peak intensities to the variables measured in the laboratory. For glucose, for example, correlations for most of the positively correlated peaks increased as is evident from the dots falling above the diagonal line. For negatively correlated metabolites most of the correlations fall near the diagonal line still producing more scatter below the diagonal line indicating increased correlations. Similar patterns are observed for the rest of the physiological variables.

## Conclusions

Normalization is an important step in MS data analysis, but it is complicated by the high complexity of biases. EigenMS has been shown to remove biases of arbitrary complexity from proteomics data [9]. Here we show that it works equally well for metabolomics data. By extension, the method should reasonably be expected to work equally well for any omics data where the variation of interest can be preserved via the fixed effects in an ANOVA model and the matrix of residuals analyzed for the presence of bias trends. The ability of EigenMS to capture complex biases and eliminate them preserves the validity of any downstream statistical analysis. The software is implemented in R and is freely available from SourceForge: http://sourceforge.net/projects/eigenms/.
